# Sofrito and Fruit Consumption Associated with Lower Risk of Type 2 Diabetes in an Urban Latin American Cohort

**DOI:** 10.3390/nu18071024

**Published:** 2026-03-24

**Authors:** Paula Calderón, Luisa Villamagua-Godoy, Verónica Cárdenas-Mazón, Martha Montalván, Rosario Suárez, Sebastián Chapela, Estefanía Bautista-Valarezo, Evelyn Frias-Toral

**Affiliations:** 1 Carrera de Nutrición y Dietética, Facultad de Ciencias de la Salud, Universidad Técnica Particular de Loja, Loja 110107, Ecuador; lmvillamagua1@utpl.edu.ec (L.V.-G.); nvcardenas1@utpl.edu.ec (V.C.-M.); 2 Escuela de Medicina, Universidad Católica de Santiago de Guayaquil, Av. Pdte. Carlos Julio Arosemena Tola, Guayaquil 090615, Ecuador; martha.montalvan@cu.ucsg.edu.ec; 3 Escuela de Medicina, Universidad de Guayaquil, Avenida 10 NO, Guayaquil 090613, Ecuador; 4 Carrera de Medicina, Facultad de Ciencias de la Salud, Universidad Técnica Particular de Loja, Loja 110107, Ecuador; rsuarez2@utpl.edu.ec (R.S.); mebautista@utpl.edu.ec (E.B.-V.); 5 Departamento de Bioquímica Humana, Facultad de Medicina, Universidad de Buenos Aires, Buenos Aires 1113, Argentina; sebachapela@gmail.com; 6 Equipo de Soporte Nutricional, Hospital Británico de Buenos Aires, Buenos Aires 1280, Argentina; 7 Escuela de Medicina, Universidad Espíritu Santo, Samborondón 092301, Ecuador; evelynft@gmail.com; 8 Division of Research, Texas State University, San Marcos, TX 78666, USA

**Keywords:** Mediterranean diet, type 2 diabetes, adherence score, protective factors, sofrito sauce

## Abstract

Objective: To evaluate the association between adherence to the Mediterranean Diet (MedDiet) and the risk of type 2 diabetes mellitus (T2D) in an urban Ecuadorian population, with emphasis on the protective role of specific dietary components and body composition. Methods: A cross-sectional analytical study was conducted with 1373 adults aged 18–75 years. Adherence to the MedDiet was assessed using the 14-item Mediterranean Diet Adherence Screener (MEDAS-14), while T2D risk was estimated using the Finnish Diabetes Risk Scale (FINDRISC). Anthropometric and body composition parameters were measured using standardized procedures. Adjusted associations between exposure variables and T2D risk were estimated using Poisson regression with robust variance, and prevalence ratios were calculated after controlling for sociodemographic and lifestyle factors. Results: Most participants showed low adherence to the MedDiet (85.2%), which was significantly associated with higher T2D risk. Multivariate analysis of individual dietary components showed relevant associations. Regular consumption of sofrito was associated with lower T2D risk (PR = 0.817; 95% CI: 0.682–0.979; *p* = 0.028). Similarly, low fruit consumption was associated with a substantially higher T2D risk (PR = 1.350; 95% CI: 1.146–1.589; *p* = 0.001). In addition, higher body mass index (BMI) and waist circumference (WC) were consistently related to greater T2D risk. Conclusions: Greater adherence to the MedDiet, particularly consumption of key components such as sofrito and fruits, was associated with lower T2D risk. These findings highlight the importance of preventive lifestyle interventions adapted to the Latin American context.

## 1. Introduction

Type 2 diabetes (T2D) is a chronic, multifactorial disease characterized by elevated blood glucose levels, mainly due to insulin resistance and pancreatic β-cell dysfunction [1]. Its prevalence has risen sharply in recent decades, becoming a major public health challenge with profound implications for quality of life and healthcare systems [2]. According to the International Diabetes Federation (IDF), T2D is influenced by both non-modifiable factors, such as age and genetic predisposition, and modifiable factors, including overweight or obesity, physical inactivity and unhealthy dietary habits [3].

Among preventive strategies, diet plays a central role. The Mediterranean diet (MedDiet) has been widely studied and identified as a dietary pattern with significant health benefits [4,5], particularly in relation to T2D prevention and management [6,7,8]. Evidence indicates that the MedDiet supports glycemic control, reduces inflammation, improves insulin sensitivity, and promotes healthy weight [6,9,10,11]. This dietary model emphasizes high consumption of plant-based foods (legumes, nuts, whole grains, vegetables, and fruits, with olive oil as the primary fat source); includes moderate intake of fish and poultry; and limits red meat, ultra-processed foods, and refined sugars [12]. Conversely, frequent consumption of industrially baked goods and sugary foods has been associated with an increased risk of T2D [13,14].

In Latin America, traditional dietary patterns also share protective features. These patterns are characterized by higher consumption of whole grains, legumes, fruits, vegetables, and tubers, combined with relatively low levels of food processing [15]. In Ecuador, for example, the traditional Andean dietary pattern includes corn, quinoa, potatoes, sweet potatoes, cassava, beans, and local fruits, providing fiber, micronutrients, and low-glycemic carbohydrates. Such patterns have been associated with a reduced prevalence of overweight and obesity, key mediators in the development of T2D [16]. However, these protective models are increasingly being displaced by greater consumption of ultra-processed foods rich in sugars and fats, reflecting the ongoing nutrition transition and its growing public health burden [17,18].

Within the MedDiet, certain components may have particularly relevant protective effects. Fruit consumption is consistently linked to a reduced risk of T2D, especially when whole fruits are consumed rather than juices, which have a higher glycemic load and lower fiber content [19,20,21]. Sofrit, a traditional Mediterranean preparation made by slowly cooking tomatoes, onions or leeks, garlic, and olive oil [22] enhances the bioavailability of carotenoids and polyphenols, compounds with antioxidant and anti-inflammatory properties [23]. Although direct evidence linking sofrito to T2D is limited, its bioactive composition suggests a potential protective role in metabolic health. Avocados, while less central to the MedDiet, are rich in monounsaturated fatty acids and have been associated with improvements in lipid profile and insulin sensitivity, although evidence for their role in T2D prevention remains less robust [24,25,26].

It is important to note that the MedDiet reflects not only specific foods but also the broader cultural and environmental context of Mediterranean populations, including active lifestyles and relatively low psychosocial stress, which together contribute to its protective effects [27,28,29]. These determinants differ substantially from those in Latin America, where rapid urbanization, socioeconomic inequalities, and the rising availability of ultra-processed foods significantly shape the T2D risk [30,31,32].

Given these contextual differences, assessing the applicability of the MedDiet in Ecuador requires evaluating both overall adherence and specific dietary components with potential protective effects. Therefore, the present study aimed to evaluate adherence to the MedDiet and its association with T2D risk in an urban Ecuadorian population, with particular focus on the role of fruit and sofrito consumption as potentially protective factors.

## 2. Materials and Methods

### 2.1. Design

This study employed a cross-sectional, analytical, observational design to evaluate T2D risk in relation to MedDiet adherence and body composition parameters. The Strengthening the Reporting of Observational Studies in Epidemiology (STROBE) guidelines were followed to ensure methodological rigor and transparent reporting.

### 2.2. Population and Sample

The sample was drawn from an urban population of 214,855 individuals residing in the city of Loja, Ecuador. The required sample size was calculated at 1218 participants, assuming an expected 15% risk of T2D, 2% precision, and a 95% confidence level. To account for an estimated 14% attrition rate, the final target sample was increased to 1388 individuals. Sample size estimation was performed using Epidat version 3.1.

**Figure 1:**
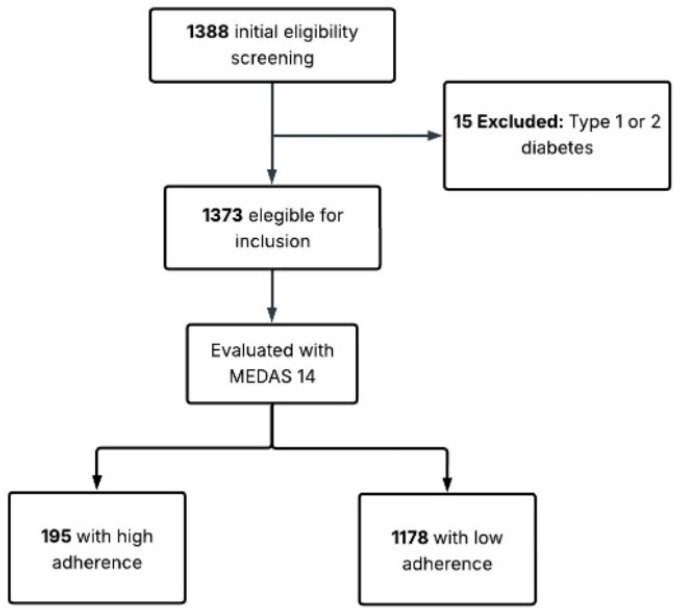
Exclusion criteria included previous T2D diagnosis, pregnancy, and cognitive impairment. Of the 1388 individuals who met the eligibility criteria, 15 with T2D were excluded, resulting in 1373.

Sampling procedure and eligibility: This study utilized a non-probability convenience sampling approach. Participant selection was based on institutional access and voluntary participation. Eligible participants were employees of public or private institutions from various sectors (academia, transportation, education, healthcare, or economics) who were affiliated with the National Social Security Institute (IESS) and aged 18 to 75 years. Invitations were disseminated through institutional human resources departments and public notices within IESS-affiliated institutions to recruit a diverse professional subset of the urban workforce. This strategy was employed to leverage established professional networks and ensure feasibility (Figure 1). The study’s emphasis on primary prevention, together with the low number of previously diagnosed T2D cases among the original participants (*n* = 15), is consistent with the “healthy worker effect,” which is typical in occupational cohorts. This further supports the usefulness of the FINDRISC tool for determining clinical risk in active but possibly underdiagnosed groups.

### 2.3. Data Collection

Data were collected using the STEPwise 3.2 questionnaire, adapted for Ecuador by the Ministry of Public Health (MSP), the National Institute of Statistics and Censuses (INEC), and the Pan American Health Organization/World Health Organization (PAHO/WHO) [33]. Information was gathered on sociodemographic characteristics, tobacco use, and alcohol consumption. Physical activity levels were assessed using the short version of the International Physical Activity Questionnaire (IPAQ) [34].

### 2.4. Anthropometric and Body Composition Assessment

Anthropometric and body composition measurements were obtained using standardized procedures and validated equipment. A multifrequency segmental analyzer (InBody 120), with a reported reliability of 98%, was used to measure body weight (kg), BMI, body fat percentage, visceral fat, skeletal muscle mass (SMM), and waist-to-hip ratio (WHR).

Height was measured to the nearest millimeter using a portable stadiometer (Seca 217), with participants standing upright in the Frankfurt horizontal plane. WC was measured at the level of the navel at the end of a normal expiration using a Cescorf measuring tape (±1 mm resolution). The manufacturers of equipment include DSM-BIA Segmental Multifrequency Body Composition Analyzer inbody 120 (InBody Co., Ltd., Eonju-ro, Gangnam-gu, Seoul 06106, Republic of Korea) and seca 217 Mobile Stadiometer (SECA, Hammer Steindamm 3-25, 22089 Hamburg, Germany).

### 2.5. Adherence to the Mediterranean Diet

Adherence to the MedDiet was assessed exclusively through the 14-item Mediterranean Diet Adherence Screener (MEDAS-14), administered in person by trained observers standardized in data collection procedures. The questionnaire evaluates nutritional intake across 14 core components, including vegetables, fruits, legumes, whole grains, nuts, and olive oil, using predefined serving-based cutoffs. No complementary dietary assessment instruments were used. MEDAS-14 is a validated instrument with a total score ranging from 0 to 14, adherence categorized as low (<9 points) or high (≥9 points) [22].

### 2.6. Risk of Type 2 Diabetes Mellitus

The FINDRISC was employed to estimate participants’ 10-year risk of developing T2D. This tool integrates eight established risk factors, including age, BMI, WC, physical activity, family history of T2D, history of impaired glycemia, antihypertensive medication use, and a single dietary item referring to daily fruit and vegetable consumption. The questionnaire was administered through standardized, observer-supervised interviews. FINDRISC has been extensively validated across European and non-European populations, including Latin America, showing moderate to good discriminative accuracy (AUC between 0.65 and 0.75) for identifying undiagnosed T2D and prediabetes [35,36,37]. Scores range from 0 to 26, with scores ≥ 12 points indicating moderate to high risk for T2D. In this study, the outcome variable was defined as moderate or greater T2D risk (FINDRISC ≥ 12) [38].

### 2.7. Ethical Approval

The study was conducted in the city of Loja, Ecuador. The study protocol was approved by the Human Research Ethics Committee (CEISH) of Hospital General San Francisco de Quito, located in Quito, Ecuador (Approval Code: 031, Approval date: 16 August 2023), which has national jurisdiction for clinical research oversight. All procedures were carried out in accordance with the Declaration of Helsinki.

### 2.8. Statistical Analysis

Data were analyzed using SPSS version 26 and EPIDAT version 3.1. Normality and homoscedasticity of variables were assessed prior to hypothesis testing. To ensure data integrity, only participants with complete information for all study instruments and key anthropometric and body composition measures were included in the final analysis. Individuals with missing data on these variables were excluded.

Descriptive statistics for categorical variables were presented as frequencies and percentages, and their associations were examined using the Chi-square test. Continuous variables were reported as means ± standard deviations (SD) or medians (interquartile range, IQR), as appropriate, and compared using Student’s *t*-test for independent samples.

#### Regression Models for Type 2 Diabetes Risk

Given the cross-sectional design and the nature of the outcome, unadjusted prevalence ratios (PRs) were first calculated to examine the association between T2D risk and MedDiet adherence. Subsequently, four multivariate regression models were constructed, using T2D risk (FINDRISC ≥ 12) as the dependent variable.

Model 1 (dietary components): This model used Poisson regression with robust variance to estimate the adjusted Prevalence Ratios (aPRs) for the individual components of the MEDAS-14 questionnaire, while controlling for the other dietary criteria included in the adherence score to isolate the independent effect of each item (e.g., sofrito consumption).

Model 2 (socio-demographic and body compositional factors): A separate model was constructed incorporating a wider range of sociodemographic and body composition variables to control for major confounding factors. This model utilized logistic regression, and model fit was assessed with the Hosmer–Lemeshow test. Covariables included educational level, marital status, alcohol and tobacco consumption, BMI, body fat percentage, visceral fat, SMM, WC and WHR.

Model 3 (dietary components and associated risk factors): This model used Poisson regression with robust variance to estimate aPRs for selected MEDAS-14 components (sofrito, fruits, and sugar-sweetened beverages), while controlling for age, sex, smoking, BMI, abdominal circumference, and muscle mass, which were identified as relevant risk factors in Models 1 and 2. To assess possible multicollinearity among the covariates, the variance inflation factor (VIF) was calculated, with VIF > 5 considered indicative of problematic collinearity.

Model 4 (MedDiet and associated risk factors): This model used Poisson regression with robust variance to estimate the aPR for overall MEDAS-14 adherence while controlling for age, sex, smoking, BMI, abdominal circumference, and muscle mass, based on the risk factors identified in Models 1 and 2. Multicollinearity was assessed using the VIF, with VIF > 5 considered indicative of problematic collinearity.

Multicollinearity assessment:

Before constructing the multivariate models, the presence of multicollinearity among explanatory variables was rigorously evaluated. Bivariate correlations among all predictors were calculated, and the VIF was estimated for each variable. Following established criteria, variables with VIF > 5 were excluded from the final adjusted models to ensure stability, reliability, and interpretability of the estimated associations.

## 3. Results

The baseline characteristics of the study participants, stratified by adherence to the MedDiet, are summarized in Table 1. Of the total cohort (*n* = 1373), the majority (85.8%) exhibited low MedDiet adherence. Individuals with high MedDiet adherence differed significantly from those with low adherence across several sociodemographic and lifestyle factors. High adherers were, on average, older (43.56 vs. 40.27 years; *p* < 0.001), more frequently female (69.7%; *p* = 0.003) and had higher educational attainment, predominantly at the undergraduate and postgraduate levels (*p* = 0.002). Regarding lifestyle characteristics, high adherence was associated with a significantly lower prevalence of smoking (8.7% vs. 14.7%; *p* = 0.025). Although alcohol consumption was lower among high adherers (50.3% vs. 53.3%; *p* = 0.429), this difference was not statistically significant.

Table 2 and Table 3 present the distribution of T2D risk score, physical activity level, and body composition measures stratified by MedDiet adherence among male and female participants. A consistent but non–significant trend was observed in both sexes, whereby lower Med Diet adherence was correlated with an elevated T2D risk score and a less favorable anthropometric profile. However, a statistically significant association was detected exclusively among women, in whom physical activity level was significantly related to MedDiet adherence (*p* < 0.05). Among women with high MedDiet adherence, physical activity level was low in 40.4%, moderate in 30.1%, and high in 29.4% of participants. In contrast, among women with low adherence, physical activity level was low in 56.7%, moderate in 29.8%, and high in 13.5% (*p* < 0.0001). The full set of stratified findings is provided in Table 2 and Table 3.

Analysis of the individual MedDiet components showed that only sofrito was associated with a significant reduction in T2D risk (PR = 0.802; 95% CI: 0.671–0.959; *p* = 0.016). In the adjusted Poisson model, after multicollinearity assessment and adjustment, participants who consumed sofrito had an 18.3% lower risk of T2D compared to those who did not (PR = 0.817; 95% CI: 0.682–0.979; *p* = 0.028). In contrast, participants who consumed carbonated or sugar-sweetened beverages had a 25% higher risk of T2D compared to non-consumers (PR = 1.247; 95% CI: 1.002–1.552; *p* = 0.048). Other MedDiet components, such as fruits, olive oil, and fish, showed a protective trend; however, these associations did not reach statistical significance (Table 4).

In men (Table 5) analysis of anthropometric and body composition measures in the unadjusted model showed that higher BMI (OR = 1.481; 95% CI: 1.373–1.596; *p* < 0.001), greater WC (OR = 1.167; 95% CI: 1.133–1.201; *p* < 0.001), and lower SMM (OR = 1.088; 95% CI: 1.041–1.137; *p* < 0.011) were associated with a higher probability of T2D risk. In the adjusted regression model, after multicollinearity tests (VIF > 5), body fat percentage, fat mass and WHR were removed. The final results showed that each unit increase in BMI was associated with a 24% higher risk of T2D (OR = 1.241; 95% CI: 1.105–1.393; *p* < 0.001). Likewise, each increase in WC was associated with an 11% greater risk of T2D (OR = 1.11; 95% CI: 1.063–1.162; *p* < 0.001). On the other hand, greater MM was associated with an 8% lower risk of T2D (OR = 0.914; 95% CI: 0.861–0.971; *p* < 0.001).

In the initial logistic regression model for men, the dependent variable was T2D risk, and the following covariates were included: educational level, marital status, tobacco and alcohol consumption, blood pressure, BMI, WC, WHR, SMM, visceral fat level (VFL), body fat percentage (BFP), and fat mass (FM). Multicollinearity was assessed using the variance inflation factor (VIF), with VIF > 5 used to identify problematic collinearity. As a result, WHR, BFP, and FM were excluded from the final model to improve the estimate stability.

In women (Table 6), the unadjusted analysis of anthropometric and body composition measures showed that higher BMI (OR: 1.328 (95% CI: 1.265–1.394), *p* < 0.001) and greater WC (OR: 1.137 (95% CI: 1.114–1.161), *p* < 0.001) were associated with T2D risk. In the adjusted regression model, after multicollinearity testing (VIF > 5), body fat percentage, fat mass, and WHR were removed. The results showed that each unit increase in BMI was associated with a 13% higher risk of T2D (OR: 1.132 (95% CI: 1.056–1.213), *p* < 0.001). Similarly, each increase in WC was associated with a 9% higher risk of T2D (OR: 1.094 (95% CI: 1.062–1.127), *p* < 0.001). Greater MM, in turn, was associated with a 7% lower risk of T2D (OR: 0.928 (95% CI: 0.873–0.987), *p* < 0.001).

In Table 7, the analysis of anthropometric factors, sociodemographic characteristics, and MedDiet components using aPRs showed that male sex was associated with a 35.4% lower probability of T2D, whereas non-smoking was associated with a 32.5% lower probability of T2D. On the other hand, higher BMI was associated with a 5.7% increase in T2D risk, and greater WC was associated with a 2.6% higher probability of developing the disease. In addition, each additional year of age was associated with a 3.6% increase in T2D risk. Regarding eating habits, not preparing sofrito with meals was associated with a 15.4% higher risk of T2D (PR: 1.154; 95% CI: 1.017–1.308; *p* = 0.026), and non-consumption of fruits was associated with a 35% higher risk of T2D (PR: 1.350; 95% CI: 1.146–1.589; *p* = 0.001).

In this generalized regression model, the dependent variable was T2D risk. The covariates included sex, tobacco use, fruit consumption, sofrito consumption, sugar-sweetened beverages intake, BMI, WC and SMM. To assess the possible multicollinearity among the covariates, VIF was calculated using a cutoff of VIF value > 5. The results showed the absence of multicollinearity.

The final comprehensive model, using Poisson regression, estimated the aPRs using the overall MEDAS-14 score as the primary dietary exposure, together with key non-dietary anthropometric and sociodemographic risk factors. The results showed that male sex, non-smoking, and high adherence to the MEDAS-14 score (≥9) were protective factors against the T2D risk (*p* < 0.05). In contrast, higher BMI and greater WC were associated with an increased likelihood of T2D risk (Table 8).

In this generalized regression model, the dependent variable was T2D risk, and the covariates included sex, tobacco use, MedDiet, BMI, WC and SMM. Multicollinearity was assessed using a VIF, with VIF > 5 considered an indicator of problematic collinearity. No evidence of multicollinearity was detected among the variables included in the model.

## 4. Discussion

In this study, the analysis of anthropometric, sociodemographic, and dietary factors revealed that male sex, non-smoking status, and high adherence to the MedDiet were associated with a lower T2D risk. This finding underscores a significant public health opportunity: improving adherence to this dietary pattern could provide meaningful benefits. Prior research has shown that each incremental point in the MEDAS score corresponds to a progressive reduction in T2D risk, with high adherence associated with up to a 50% lower incidence of the disease, mediated by enhanced insulin sensitivity, improved lipid metabolism, and reduced systemic inflammation [39]. However, the low adherence found in this study is consistent with observations from other Latin American populations [40,41,42], where cultural and environmental barriers hinder the adoption of the MedDiet pattern. Nevertheless, traditional Latin American diets, such as corn-, legume-, and vegetable-based diets in Mexico; rice- and- bean patterns in Costa Rica; and complex carbohydrate-based diets in Bolivia, share core principles of the MedDiet and have shown protective effects against metabolic disorders [43,44,45,46]. Ecuador, however, has undergone a marked nutritional transition, with rural T2D prevalence reaching 20.4% and urban low-income communities showing high rates of obesity and central adiposity, driven by diets poor in fruits and vegetables and high in salt and sugar [47,48].

Regarding specific dietary habits, this study has shown that not preparing sofrito as part of meals was associated with a 15.4% higher risk of T2D, whereas not consuming fruits increased the risk by 35%. Different studies have shown that fruit-based beverages and purees may exert favorable metabolic and vascular effects, including reduced T2D risk in genetically predisposed populations, low glycemic responses, and improved vascular function and muscle maintenance under training conditions [49,50,51]. Valder et al. suggest that regular consumption of polyphenol-rich or low-glycemic fruit preparations may contribute to better metabolic health [49]. Sofrito does not have a standardized recipe or a fixed quantitative composition, as its formulation varies across households, regions, and culinary contexts. In the MEDAS-14 survey, consumption frequency was collected using a dichotomous response, and our analysis was therefore based on the reported presence of the dish in the diet rather than on quantification of its individual components. Furthermore, there is currently limited scientific evidence precisely describing the quantities of onion, garlic, oil, and tomato used in sofrito preparation across domestic contexts, which constitutes a relevant area for future research. However, DiFrancesco et al. suggested that, for approximately eight servings of rice (two cups), sofrito can be prepared with one small onion, four cloves of garlic, one small red bell pepper, 1/4 cup of oil, and 1/4 cup of blended tomato [49,50,51].

The increasing consumption of ultra-processed foods (UPFs) and the decline in home cooking further distance current dietary habits from the MedDiet model [51,52,53,54]. Although our initial data indicated that participants who consumed carbonated or sugar-sweetened beverages, classified as UPFs, had a 25% higher risk of T2D compared with non-consumers, this association lost statistical significance in the multivariate analysis. Within the MEDAS framework, this behavior represents a marker of low adherence to the MedDiet pattern, characterized by minimal consumption of added sugars and a preference for water or natural beverages. The observed association therefore reinforces the internal consistency of the MEDAS construct, in which items reflecting poor-quality dietary practice, such as regular consumption of sugary drinks, diminish the overall protective potential of the diet. These findings are consistent with extensive evidence linking high intake of sugar-sweetened beverages to adverse metabolic outcomes [55,56,57]. In the Latin American region, where consumption of these beverages remains particularly high, the population-attributable burden of disease is substantial. In Mexico, 19% of deaths from T2D, cardiovascular disease, and obesity-related cancers are attributable to sugary beverages, with the greatest impact among younger adults and low-income groups [55]. Similar trends have been observed in various Latin American countries [30,56], representing a major public health concern in the region and highlighting the need for preventive strategies.

Higher consumption of sofrito (≥2 servings/week) and fruit (≥3 servings/day) was independently associated with a lower risk of T2D. These associations remained significant after multivariate adjustment, indicating that individual components of the MEDAS score can exert meaningful metabolic effects even when overall adherence is low. From a mechanistic perspective, these components may act synergistically within the MedDiet pattern to enhance glycemic control and reduce oxidative stress.

The protective association observed for sofrito intake is consistent with the biological plausibility of the MedDiet model, in which tomato-based dishes prepared with olive oil and vegetables contribute to a high density of bioactive compounds. Sofrito, typically prepared with tomato, onion, garlic, and oil, is rich in carotenoids, lycopene, organosulfur compounds, and other bioactive molecules with anti-inflammatory and antioxidant properties. Lycopene, the main carotenoid in tomatoes, helps reduce oxidative stress and lipid peroxidation, both of which can impair insulin signaling and glucose metabolism [57,58]. Polyphenols from olive oil and vegetables exert anti-inflammatory effects by inhibiting pro-inflammatory cytokines and modulating pathways such as NF-κB and PPARγ, which are closely related to glucose metabolism and insulin action [59,60,61]. Additionally, organosulfur compounds from garlic and onion may display hypoglycemic effects by enhancing insulin secretion and protecting pancreatic β-cells, and reducing systemic inflammation [62,63,64]. Collectively, these mechanisms align with the broader biological rationale of the MedDiet, in which the synergistic interaction of multiple plant-derived foods and healthy fats contributes to improved metabolic outcomes and lower T2D.

Although beneficial elements of the MedDiet, such as sofrito, are present in Ecuadorian cuisine, their potential protective effects may be diminished by dietary patterns dominated by refined oils, flours, and processed carbohydrates, which elevate glycemic load and worsen lipid profiles. Unlike the MedDiet, which prioritizes extra virgin olive oil as a source of monounsaturated fats and polyphenols, Ecuadorian households commonly use refined sunflower, soybean or palm oils [65], which lack many of the bioactive compounds responsible for olive oil’s cardioprotective effects [65].

In the multivariate models, higher BMI and WC were consistently associated with an increased risk of T2D, whereas greater SMM was independently associated with a reduced risk. This protective association was distinct and statistically significant in sex-specific adjusted models that rigorously controlled for adiposity-related factors. These findings reinforce the well-established role of body composition as a major determinant of metabolic health [66].

Regarding sex differences, male sex appeared to be a protective factor for T2D in this study. These findings align with prior research showing that fat distribution modulates metabolic risk, with WC differences often more pronounced in women and visceral fat differences greater in men [66]. Hormonal and physiological differences, particularly the divergent effects of testosterone and estradiol on fat deposition and muscle maintenance, may explain these disparities [67].

Conversely, greater skeletal muscle mass was associated with lower T2D risk, in sex-specific adjusted logistic regression models, but this association was substantially attenuated in the final comprehensive models after adjustment for the strong confounding effects of BMI and WC. This attenuation does not negate the biological protective role of MM; rather, it suggests a profound epidemiological principle: the risk-modifying effect of SMM may be mediated by the absolute level of adiposity. Individuals with higher adiposity may appear to have greater muscle size, but that muscle may be less functional because of fatty infiltration [66]. Moreover, individuals with sarcopenic obesity exhibit a higher risk of T2D than those with obesity or sarcopenia alone [68]. These observations are consistent with prior evidence demonstrating that the MedDiet pattern contributes to preservation of muscle mass and reduction in visceral fat [66,68,69,70]. From a public health perspective, these findings suggest that interventions aimed to improve adherence to the MedDiet could simultaneously target adiposity reduction and muscle preservation, thereby amplifying their impact on metabolic health and T2D prevention.

Our analysis also showed no significant differences in anthropometric indicators (BMI, WC, WHR, body fat and visceral fat) between participants with low versus high MedDiet adherence. However, physical activity displayed a clear relationship with dietary quality: women with higher levels of physical activity were more likely to adhere to the MedDiet, a pattern consistent with evidence indicating that physically active individuals tend to score higher on MEDAS due to greater health awareness and behavioral self-regulation [71,72,73]. This synergy between dietary and physical activity patterns reinforces the holistic nature of the Mediterranean lifestyle, in which diet and movement jointly contribute to metabolic resilience.

Sociodemographic factors were also strongly associated with T2D. Older participants exhibited a higher risk, consistent with the findings of Pan et al., who described that individuals with greater biological age and higher genetic risk showed a markedly higher incidence of T2D than those with lower biological age and genetic risk [74]. Educational attainment also appeared relevant [75]; participants with university or postgraduate education had significantly higher adherence, consistent with the literature linking education to greater health literacy and a greater ability to make informed choices [27,76,77,78,79]. Studies in Ecuador have documented a double burden of malnutrition in rural areas, where non-native women more frequently purchase UPFs, while native women rely predominantly on home-produced foods, although they still suffer from micronutrient deficiencies. Dietary diversity remains limited and is strongly influenced by education, income, and food insecurity [80,81].

Lifestyle factors followed similar trends. Our analysis confirmed non-smoking status as a strong protective factor against T2D after multivariate adjustment. Chi et al., demonstrated that smoking status was associated with a higher risk of T2D, coronary artery disease, myocardial infarction, and heart failure [82]. This concordance reinforces the hypothesis of shared biological mechanisms linking smoking to insulin resistance.

This study presents several methodological strengths. It was conducted according to STROBE guidelines and included a large, well-powered sample (*n* = 1373), enhancing the reliability of the findings. The use of validated instruments, MEDAS-14 to assess dietary adherence, FINDRISC to estimate T2D risk, and IPAQ for physical activity, alongside standardized anthropometric and body composition measurements, including SMM and visceral fat via InBody120, ensured the collection of high-quality, multidimensional data. Importantly, the instruments were contextually adapted for the Ecuadorian population, increasing local relevance, and the use of multivariate models allowed for adjustment across sociodemographic, lifestyle, and physiological factors.

Several limitations should also be acknowledged. First, the non-random sampling approach may limit generalizability to the broader population, although participants were recruited from diverse occupational sectors. Second, the cross-sectional design prevents causal inference and raises the possibility of reverse causality. Third, reliance on self-reported questionnaires introduces the potential for recall and social desirability bias. Although both FINDRISC and MEDAS-14 include items on fruit and vegetable consumption, FINDRISC only captures a single dichotomous item on daily intake, whereas MEDAS-14 provides a broader assessment of dietary components and portion-based thresholds. In this study, FINDRISC was used strictly as a validated endpoint for estimating T2D risk, while MEDAS-14 served to characterize dietary patterns.

Additionally, the imbalance between low- and high-adherence groups when applying the standard MEDAS-14 cut-off (<9 vs. ≥9 points) may have reduced statistical power in the high-adherence group. Nevertheless, this cutoff follows validated criteria [27,83] and reflects the true dietary distribution of the population, in which low adherence has been documented in other Latin American settings, including the ELANS study across eight countries [84], and among Colombian university students [41].

Finally, although MEDAS-14 is validated and widely used in Mediterranean and non-Mediterranean contexts, it is a simple tool that captures dietary type, frequency, and approximate quantity without gram-level detail. Its use in Ecuador may therefore not fully reflect cultural and contextual dietary practices, which could affect the precision of dietary exposure measurement. In this cohort of workers aged 18 to 75 (mean: 41.9 years), the low prevalence of pre-existing T2D (about 1%) may seem lower than total national estimates, but it is representative of an urban working population. Importantly, our study revealed that a significant proportion of this “apparently healthy” group already had a high 10-year risk of T2D, underscoring the need for early screening in Latin American urban settings. Regarding alcohol consumption, this variable was assessed only as a lifetime yes/no variable, without capturing type, frequency, or quantity, which limits interpretation, particularly in the context of the Mediterranean diet, where moderate wine consumption is traditionally considered a component. Therefore, our findings regarding alcohol should be interpreted with caution. Furthermore, unmeasured confounders such as stress, sleep quality, and genetic predisposition were not assessed. Future studies in Latin America should incorporate longitudinal designs and more detailed dietary assessment tools, including quantitative food frequency questionnaires and biomarker-based measures, to strengthen causal inference and improve exposure accuracy.

## 5. Conclusions

This cross-sectional investigation identified context-specific determinants of T2D risk in an urban Ecuadorian cohort characterized by predominantly low adherence to the MedDiet pattern. Overall adherence to the MedDiet, after rigorous adjustment for key anthropometric and lifestyle confounders, demonstrated a statistically significant protective association with T2D risk. Disaggregated analysis revealed strong associations tied to specific dietary components and physiological factors; notably, consumption of sofrito and fruits was independently associated with a significantly lower prevalence ratio of T2D risk. In contrast, elevated BMI and WC emerged as the most consistent and robust determinants of heightened T2D risk across all models, whereas greater SMM was independently associated with a lower risk in sex-specific models. Additionally, male sex and non-smoking status were identified as significant protective factors, whereas older age was associated with greater risk. These findings underscore the critical value of promoting culturally grounded and targeted dietary interventions, such as increasing sofrito and fruit consumption. Furthermore, public health strategies in Latin American populations may benefit from a dual focus on improving body composition by reducing central adiposity while preserving or increasing metabolically active muscle mass. Longitudinal research is warranted to confirm the causal pathways suggested by these associations.

## Figures and Tables

**Table 1:** Association between MedDiet adherence and sociodemographic parameters.

		Adherence to MedDiet		*p* Value
		High 195 (14.2%)	Low 1178 (85.8%)	
Age, years	Mean (SD)	43.56 (10.2)	40.27 (10.11)	0.000 ***
Gender (*n*, %)	Male	59 (30.3%)	490 (41.6%)	0.003 **
	Female	136 (69.7%)	688 (58.4%)	
Education (*n*, %)	Elementary school	3 (1.5%)	41 (3.4%)	0.002 **
	High school	23 (11.8%)	195 (16.6%)	
	College	98 (50.3%)	667 (56.6%)	
	Graduate	71 (36.4%)	275 (23.3%)	
Current smoker (*n*, %)	No	178 (91.3%)	1005 (85.3%)	0.025 *
	Yes	17 (8.7%)	173 (14.7%)	
Alcohol consumption, last 30 days (*n*, %)	No	97 (49.7%)	550 (46.7%)	0.429
	Yes	98 (50.3%)	628 (53.3%)	

* *p* value < 0.05; ** *p* value < 0.01; *** *p* value < 0.001; Student’s *t* test and Chi-squared test.

**Table 2:** Body composition according to MedDiet Adherence, males.

		Adherence to MedDiet		*p* Value
		High Adherence 59 (10.7%)	Low Adherence 490 (89.3%)	
T2D risk score	<12 points	46 (78.0%)	333 (68.0%)	0.116
	≥12 points	13 (22.0%)	157 (32.0%)	
PA level	Low	17 (28.8%)	184 (37.6%)	0.251
	Moderate	24 (40.7%)	150 (30.6%)	
	High	18 (30.5%)	156 (31.8%)	
BMI	Mean (SD)	27.4 (3.0)	27.7 (4.2)	0.566
WC	Mean (SD)	94.9 (9.4)	95.1 (11.2)	0.885
WHR	Mean (SD)	0.926 (0.03)	0.928 (0.04)	0.803
SMM	Mean (SD)	31.2 (4.6)	30.8 (4.2)	0.496
BFP	Mean (SD)	27.9 (6.8)	28.7 (7.2)	0.420
VFL	Mean (SD)	9.1 (3.7)	9.5 (4.3)	0.534

PA: physical activity; BMI: body mass index, WC: waist circumference; WHR: waist-to-hip ratio; SMM: skeletal muscle mass; BFP: body fat percentage; VFL: visceral fat level; SD: Standard Deviation.

**Table 3:** Body composition according to MedDiet Adherence, females.

		Adherence to MedDiet		*p* Value
		High Adherence 136 (16.5%)	Low Adherence 668 (83.5%)	
T2D risk score	<12 points	87 (64.0%)	408 (59.3%)	0.310
	≥12 points	49 (36.0%)	280 (40.7%)	
PA level	Low	55 (40.4%)	390 (56.7%)	0.000 *
	Moderate	41 (30.1%)	205 (29.8%)	
	High	40 (29.4%)	93 (13.5%)	
BMI	Mean (SD)	26.6 (3.7)	27.2 (4.4)	0.180
WC	Mean (SD)	87.4 (9.1)	88.6 (10.8)	0.215
WHR	Mean (SD)	0.91 (0.05)	0.92 (0.05)	0.379
SMM	Mean (SD)	20.9 (2.7)	21.3 (3.1)	0.195
BFP	Mean (SD)	38.7 (5.8)	39.1 (6.3)	0.476
VFL	Mean (SD)	11.9 (3.7)	12.3 (4.1)	0.331

PA: physical activity; BMI: body mass index; WC: waist circumference; WHR: waist-to-hip ratio; SMM: skeletal muscle mass; BFP: body fat percentage; VFL: visceral fat level; SD: Standard Deviation; * *p* value < 0.001.

**Table 4:** Unadjusted and Multivariable Adjusted Prevalence Ratios (PRs) of Type 2 Diabetes Risk Associated with Individual MedDiet Components.

	T2D Risk (FINDRISC < 12)	T2D Risk (FINDRISC > 12)	Unadjusted		Adjusted	
	*n* = 874 F (%)	*n* = 499 F (%)	PR (95% CI)	*p* Value	PR (95% CI)	*p* Value
MedDiet Component						
Olive oil	302 (67.4%)	146 (32.6%)	0.854 (0.704–1.036)	0.109	0.868 (0.711–1.059)	0.163
Vegetables ^a^	182 (68.2%)	85 (31.8%)	0.850 (0.673–1.074)	0.174	0.889 (0.697–1.133)	0.341
Fruits ^a^	212 (69.3%)	94 (30.7%)	0.809 (0.647–1.013)	0.065	0.821 (0.654–1.032)	0.091
Processed or red meats ^a^	588 (62.6%)	351 (37.4%)	1.096 (0.905–1.328)	0.349	1.082 (0.889–1.317)	0.433
Butter or cream or margarine ^a^	670 (63%)	394 (37%)	1.090 (0.879–1.351)	0.434	1.028 (0.823–1.284)	0.805
Carbonated or sugary Beverages ^a^	621 (61.8%)	384 (38.2%)	1.223 (0.993–1.506)	0.059	1.247 (1.002–1.552)	0.048 *
Wine ^a^	59 (67.8%)	28 (32.2%)	0.879 (0.600–1.287)	0.506	0.982 (0.665–1.448)	0.925
Legumes ^a^	523 (64.8%)	284 (35.2%)	0.926 (0.776–1.106)	0.398	0.960 (0.803–1.149)	0.660
Fish ^a^	112 (69.1%)	50 (30.9%)	0.832 (0.622–1.115)	0.219	0.878 (0.654–1.180)	0.389
Pastries. cakes. cookies or pasta ^a^	517 (62.2%)	309 (37.4%)	1.077 (0.899–1.290)	0.421	1.069 (0.888–1.286)	0.480
Nuts ^a^	381 (65.1%)	204 (34.9%)	0.931 (0.779–1.113)	0.436	0.969 (0.805–1.167)	0.742
Preference for white meat ^a^	723 (63.6%)	413 (36.4%)	0.993 (1.333–0.962)	0.962	0.991 (0.782–1.255)	0.938
Sofrito sauce ^a^	593 (66.6%)	298 (33.4%)	0.802 (0.671–0.959)	0.016 *	0.817 (0.682–0.979)	0.028 *

T2D: Type 2 Diabetes; FINDRISC: Finnish Diabetes Risk Scale; * *p* < 0.05; PR: prevalence ratio. The multivariable adjusted model examine the T2D risk association to variables like ^a^ ≥2 servings/day of vegetables; ≥3 servings/day of fruit; <1/day red or processed meats; <1/day butter or margarine or cream; >1/day carbonated or sugary beverages; ≥7 glasses/week of wine; ≥3 servings/week of legumes; ≥3 servings/week of fish; >2/week industrial pastries. cakes. cookies or pasta; ≥3 servings/week of nuts; ≥2 servings/week of sofrito sauce with oil and tomato, garlic, onion or leek. The scoring criteria for the MEDAS-14 are defined.

**Table 5:** Association of Risk Factors with Type 2 Diabetes Risk (Unadjusted and Adjusted ORs) in Males.

	Unadjusted		Adjusted	
	OR (95% CI)	*p* Value	OR (95% CI)	*p* Value
BMI	1.481 (1.373–1.596)	<0.001	1.303 (1.122–1.513)	<0.001 **
WC	1.167 (1.133–1.201)	<0.001	1.129 (1.068–1.193)	<0.001 **
SMM	1.088 (1.041–1.137)	<0.001	0.907 (0.849–0.970)	0.004 *

BMI: Body mass index; WC: Waist circumference; SMM: Skeletal muscle mass * *p* value < 0.01; ** *p* value < 0.001; Odds Ratio and Logistic Regression.

**Table 6:** Association of Risk Factors with Type 2 Diabetes Risk (Unadjusted and Adjusted ORs) in Females.

	Unadjusted		Adjusted	
	OR (95% CI)	*p* Value	OR (95% CI)	*p* Value
BMI	1.328 (1.265–1.394)	<0.001	1.132 (1.056–1.213)	<0.001 **
WC	1.137 (1.114–1.161)	<0.001	1.094 (1.062–1.127)	<0.001 **
SMM	1.107 (1.056–1.161)	<0.001	0.928 (0.873–0.987)	0.018 *

BMI: Body mass index; WC: Waist circumference; SMM: Skeletal muscle mass * *p* value < 0.05; ** *p* value < 0.001; Odds Ratio and Logistic Regression.

**Table 7:** Comprehensive model for a combination of anthropometric, sociodemographic, and key individual dietary factors (Fruits, Sofrito, Carbonated or sugar-sweetened beverages).

	*p* Value	PR	95% CI	
			Inferior	Superior
Sex, (male)	*** <0.001	0.646	0.514	0.812
Smoking status (Non-smokers)	** 0.009	0.798	0.675	0.944
BMI	*** <0.001	1.057	1.034	1.080
WC	*** <0.001	1.026	1.015	1.036
SMM	0.897	0.999	0.979	1.019
Age	*** <0.001	1.036	1.029	1.043
Fruit consumption (Non-consumers)	*** <0.001	1.350	1.146	1.589
Sofrito intake (Non-consumption)	* 0.026	1.154	1.017	1.308
SSBs intake (Consumers)	0.841	1.015	0.877	1.175

BMI: Body mass index; WC: Waist circumference; SMM: Skeletal muscle mass; SSBs: Sugar-Sweetened Beverages; * *p* value < 0.05; ** *p* value < 0.01; *** *p* value < 0.001; Prevalence ratio, Poisson regression.

**Table 8:** Comprehensive model using the overall Mediterranean Diet score as the primary dietary exposure, alongside key non-dietary risk factors.

	*p* Value	PR	95% CI	
			Inferior	Superior
Sex (male)	*** <0.001	0.658	0.526	0.823
Smoking (Non-Smoking)	* 0.015	0.814	0.690	0.961
BMI	*** <0.001	1.057	1.035	1.079
WC	*** <0.001	1.026	1.016	1.037
SMM	0.688	0.996	0.976	1.016
Age	*** <0.001	1.035	1.028	1.043
MEDAS-14 score	** 0.001	0.947	0.918	0.978

BMI: Body mass index; WC: Waist circumference; SMM: Skeletal muscle mass * *p* value < 0.05; ** *p* value < 0.01; *** *p* value < 0.001; Prevalence ratio, Poisson regression.

## Data Availability

The original contributions presented in this study are included in the article. Further inquiries can be directed to the corresponding author.
